# Platinum Wire-Embedded Culturing Device for Interior Signal Recording from Lollipop-Shaped Neural Spheroids

**DOI:** 10.34133/cbsystems.0220

**Published:** 2025-03-05

**Authors:** Hongyong Zhang, Nan Huang, Sumin Bian, Mohamad Sawan

**Affiliations:** ^1^ CenBRAIN Neurotech, School of Engineering, Westlake University, Hangzhou, Zhejiang 310030, China.; ^2^ School of Life Science, Westlake University, Hangzhou, Zhejiang 310030, China.

## Abstract

Three-dimensional (3D) neural cultures are increasingly recognized for their complexity and resemblance to in vivo neural microenvironments. In this paper, we present a novel 3D cell culturing and noninvasive characterization technique of neural spheroids. Based on embedded platinum wires, the cultured cells are lollipop-shaped spheroids where axons are extended and integrated around the embedded wires. Electrical microstimulation enhanced the connectivity between spheroids and demonstrated signal propagation among them. The resultant axonal elongation facilitated the formation of robust neural tracts interconnecting the neural spheroids. Variation of cells’ density allows to adjust the spheroid’s diameter, identifying 1 million cells as good number of cells for robust spheroid formation. Recordings of spheroid activities reveal higher-quality neural signal measurement from interior cells compared to those obtained from exterior cells. Viability assays confirmed the efficacy of the proposed culturing technique for sustained growth of neural spheroids over a 1-month period. The proposed spheroid culturing technique holds potential applications in various fields, such as development of brain organoids, which enables real-time interconnection characterization and sensing of environment conditions.

## Introduction

The remarkable capacity of the human brain to perform complex functions relies on the integration and coordination of neural circuits across various cortical regions [1,2]. At the core of this intricate network are interregional cortical tracts, which serve as fundamental architectural elements for connecting and orchestrating neural activity between distinct brain regions [3]. These tracts are composed of axons that extend across regions, forming bundles known as cerebral tracts within the white matter [4]. Extensive research on brain anatomy and connectivity, known as connectome studies, has revealed that a important portion of axons in cerebral tracts establish reciprocal connections between 2 cortical regions [5,6]. Specifically, association tracts are responsible for reciprocal interconnections within a single cerebral hemisphere [7]. Understanding the mechanistic significance of these interregional projections in the development of neural circuits is a crucial step toward unraveling the mysteries of brain function [8,9]. Despite the anatomical and functional significance of cerebral tracts, our understanding of the underlying mechanisms governing their formation and organization remains limited [10,11]. This limitation primarily stems from the absence of an effective in vitro model system. However, in recent years, the field of in vitro neural tissue modeling has undergone substantial advancements, providing researchers with powerful tools to investigate the complexities of neural circuitry [12,13]. For instance, Osaki et al*.* [14] explored an in vitro neural tissue model for interregional connections, connecting 2 cerebral organoids using a bundle of reciprocally extended axons. The interconnected organoids displayed a more intricate and robust oscillatory activity compared to conventional or directly fused cerebral organoids, indicating that axonal connections between organoids augment and sustain complex network behaviors. These groundbreaking studies herald the advent of novel pathways for elucidating the intricacies of the human brain.

Neural spheroids, which are three-dimensional (3D) cellular aggregates comprising neural cells (NCs), have emerged as powerful tools in neuroscience research [15]. These self-assembled structures mimic the complexity and functionality of in vivo neural tissues, making them valuable models for studying neuronal development, disease mechanisms, and potential therapeutic interventions [16]. Furthermore, neural spheroids offer an unprecedented platform for examining the intricate workings of neural circuits within a controlled environment [17]. The ability to record neural signals directly from within these spheroids presents an unparalleled opportunity to gain profound insights into the complex dynamics of neural networks at a cellular level. Conversely, traditional extracellular recordings using microelectrode arrays (MEAs) have permitted researchers to investigate neuronal activity at the population scale [18–20]. Additionally, some 3D electrodes have been developed to record signals from neural spheroids [21,22]. For example, Huang et al. [23] developed miniaturized wafer-integrated MEA caps for organoids and neural spheroids, composed of self-folding polymer leaflets with conductive polymer-coated metal electrodes. The tunable folding of the minicapes’ polymer leaflets, guided by mechanics simulations, facilitates versatile recording capabilities from organoids of varying sizes. Nevertheless, limitations in spatial resolution and the inability to capture signals emanating from interior neurons have limited our understanding of the intricate mechanisms underlying neural connectivity.

Human neural spheroids successfully mimic a substantial portion of the cellular diversity and developmental anatomy of the human brain, yet the intricate physiology of neuronal circuits within these spheroids remains largely uncharted [24]. Current prevalent approaches for signal recording within neural spheroids involve either placing sliced spheroids onto 2D MEA [25] or inserting 3D needle electrode arrays directly into the spheroid [26]. For instance, Kim et al. [27] introduced an innovative pressure-sensitive transistor array equipped with integrated 3D liquid-metal electrodes, which were implanted into cardiac organoids to capture internal signals. However, these invasive techniques pose significant risks to neurons, potentially disrupting cellular electrophysiological activities and hindering the study of neural function, including interspheroid communication. The rigidity required of implanted electrodes to avoid bending or breakage during insertion often leads to cellular damage and compromised neuron–electrode interfaces, especially in environments involving slight vibrations or fluid dynamics. In an effort to mitigate these issues, noninvasive optical imaging methods have been proposed [28], but these techniques are hampered by limited penetration depth, reduced sensitivity, and temporal resolution, which subsequently restricts their effectiveness. Thus, there is a pressing need for the development of advanced recording techniques that can balance minimal invasiveness with high sensitivity and resolution, thereby facilitating a deeper and more comprehensive understanding of the intricate neuronal circuits within human neural spheroids.

Building upon recent advancements in neuroscience, we introduce an innovative in vitro neural spheroid model that is tailored to investigate interregional neural connections. This model incorporates a novel wire-embedded device that supports the culture and growth of neural spheroids in a controlled environment. A key feature of this device is the integration of a platinum (Pt) wire, which enables the noninvasive recording of neural signals from within the spheroids without compromising their structural integrity. This approach maximizes the preservation of the delicate neural networks within the spheroids while allowing for high temporal resolution recording of neural signals. The pre-embedded Pt wire ensures minimal disruption to the neural networks, thereby preserving their natural function and connectivity. Compared to existing techniques, our device offers several advantages. First, it minimizes damage to neurons, which is crucial for accurate and reliable neural signal recording. Second, it exhibits high sensitivity, enabling the detection of subtle changes in neural activity. Lastly, it is relatively easy to obtain and use, making it accessible to a wider range of researchers. This groundbreaking technology provides unprecedented access to the neural microenvironment, allowing us to explore the dynamics of individual neurons and their interactions within the spheroids. Furthermore, our device enables the manipulation and connection of multiple spheroids, creating a network of interconnected neural spheres. This capability facilitates a comprehensive exploration of neural network dynamics, offering new insights into the functioning of the brain and potential therapeutic interventions.

**Fig. 1.**
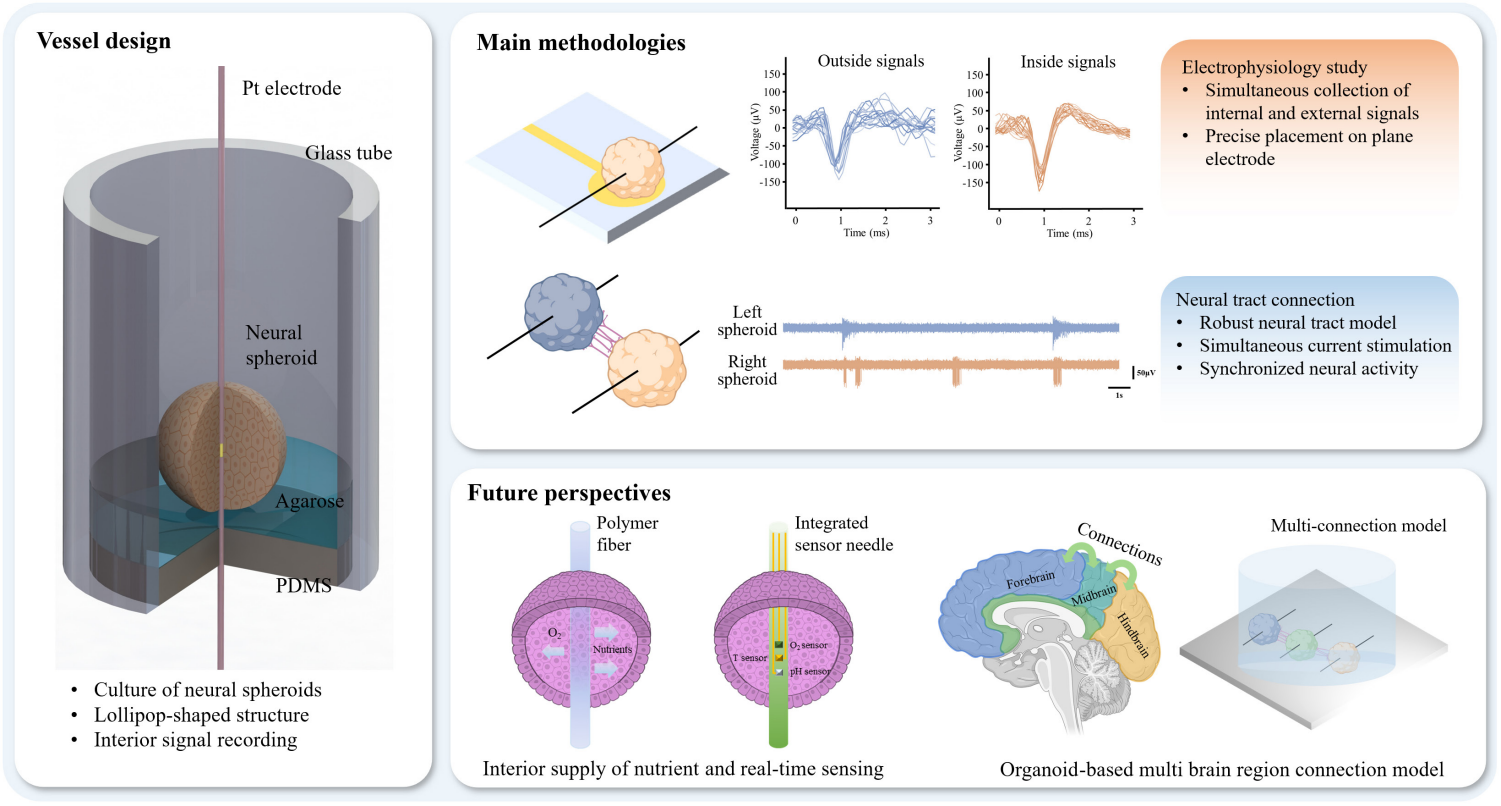
Schematic representation of the wire-embedded 3D neural spheroid culture device, consisting of glass tube, agarose, PDMS, and Pt wire electrode. The agarose provided a low adsorption surface to form neural spheroid, and Pt wire electrode was pre-inserted to obtain interior signal recording without damage. The device allows for electrophysiology study from both interior and exterior of the neural spheroid and building neural tract connection model by employing simultaneous fading sinusoidal signals to stimulate 2 close neural spheroids. This platform has the potential to be expanded for use in various applications, including the internal supply of nutrients, real-time sensing, and organoids-based multi-brain region connection models.

In this work, we designed a wire-embedded 3D neural spheroid culture device that not only supports the culture and growth of neural spheroids but also facilitates real-time monitoring of both interior and exterior neural signals without damage (Fig. 1). This is achieved by the pre-insertion of Pt wire and the placement of microelectrodes on the bottom. Furthermore, we employed simultaneous fading sinusoidal signals to stimulate the neural spheroids, thereby enhancing their interconnection and synchronization, forming robust neural tract connections. Our study leverages neural spheroids as a powerful tool for exploring neural connection dynamics and potential therapeutic strategies.

## Materials and Methods

### Neural stem cell culture

Human-induced pluripotent stem cells (hiPSCs) were utilized to derive human neurons following the neural induction protocol provided by STEMCELL Company (Shanghai, China). Initially, hiPSCs were induced into neural progenitor cells (NPCs) using the STEMdiff SMADi Neural Induction Kit (catalog #08581). Subsequently, NPCs were differentiated into NCs with STEMdiff Forebrain Neuron Differentiation Basal Medium (catalog #08601). To induce neuronal maturation and functionality, the STEMdiff Neuron Maturation Kit (catalog #08510) was employed. The hiPSC line used in this study was obtained commercially from Sanqi Biological Inc. (Shenzhen, China).

### Immunostaining

Neurons were fixed with 4% paraformaldehyde (PFA) for 10 min, followed by permeabilization with 0.3% Triton X-100 in phosphate-buffered saline (PBS) for 30 min. To prevent nonspecific binding, cells were blocked overnight at 4 °C in a solution containing PBS with 3% bovine serum albumin and 0.1% Triton X-100. Primary antibodies were added to the blocking solution and incubated at room temperature for 2 h. Similarly, secondary antibodies were applied under the same conditions and incubated for 1 h. The stained samples were sealed with Fluoromount Aqueous Mounting Medium. Specific markers for neurons, including TUJ1 (beta III tubulin, 1:1,000, Abcam, UK) and TAU (1:1,000, Cell Signal Technology, USA), were used, along with DAPI (4′,6-diamidino-2-phenylindole; 1:500, Invitrogen, USA) as a nuclear marker. Neuronal maturation was observed under a fluorescence microscope.

### Acquisition and analysis of neural signals

Neural signals inside and outside the neural spheroids were recorded using a commercial recording system, OmniPlex Neural Recording Data Acquisition System (Plexon Inc., USA). Data acquisition was performed with a gain of 1,000× and a bandpass filter ranging from 200 Hz to 4 kHz to optimize signal capture and reduce noise interference. The sampling rate was set at 30 kHz, taking into account the approximate duration of spikes being around 2 ms. This frequency is more than sufficient to accurately capture and record the signals, ensuring minimal loss of information.

For spike detection, the “relative threshold method” is employed. This method dynamically adjusts the threshold as a fraction or multiple of the baseline signal, fostering autonomy in adapting to the unique characteristics of diverse signals and minimizing human intervention. Referring to the relevant neural recording literature [23] and our own raw data, spike detection and analysis were carried out using the accompanying software, identifying spikes as signals exceeding a threshold of 6 standard deviations from the mean noise level. Regarding bursts detection, a distinctive hallmark of mature neural cultures cultivated in vitro is the synchronized and intense spiking activity, collectively referred to as bursts [29]. Referring to some authoritative articles [21], bursts were defined as a minimum of 5 consecutive spikes with a maximum interspike interval of 100 ms.

### Scanning electron microscopy

For scanning electron microscopy (SEM) analysis, neural spheroids were fixed in a solution containing 2.5% glutaraldehyde and 2% PFA (Yuanye Biotechnology, Shanghai, China) at 4 °C for 1 h. The fixed sample was washed 3 times with 0.1 M phosphate buffer (PB) (pH 7.3) to remove excess fixative. Secondary fixation involved immersing the neurons in 1% osmic acid (dissolved in 0.1 M PB) on ice for 1 h. Afterward, the sample was washed 3 times with deionized water to eliminate residual osmic acid. To facilitate SEM analysis, the sample was dehydrated using a gradient of alcohol concentrations, starting from 30% and gradually increasing to 95%, followed by 3 subsequent dehydration steps with 100% alcohol. The dehydrated sample was then critically dried to ensure complete dryness while preserving its structure. Finally, a thin layer of approximately 5-nm Pt coating was applied to enhance the sample’s conductivity and optimize imaging during SEM analysis.

## Results

### Generation of neural spheroids with Pt wire inserted

**Fig. 2.**
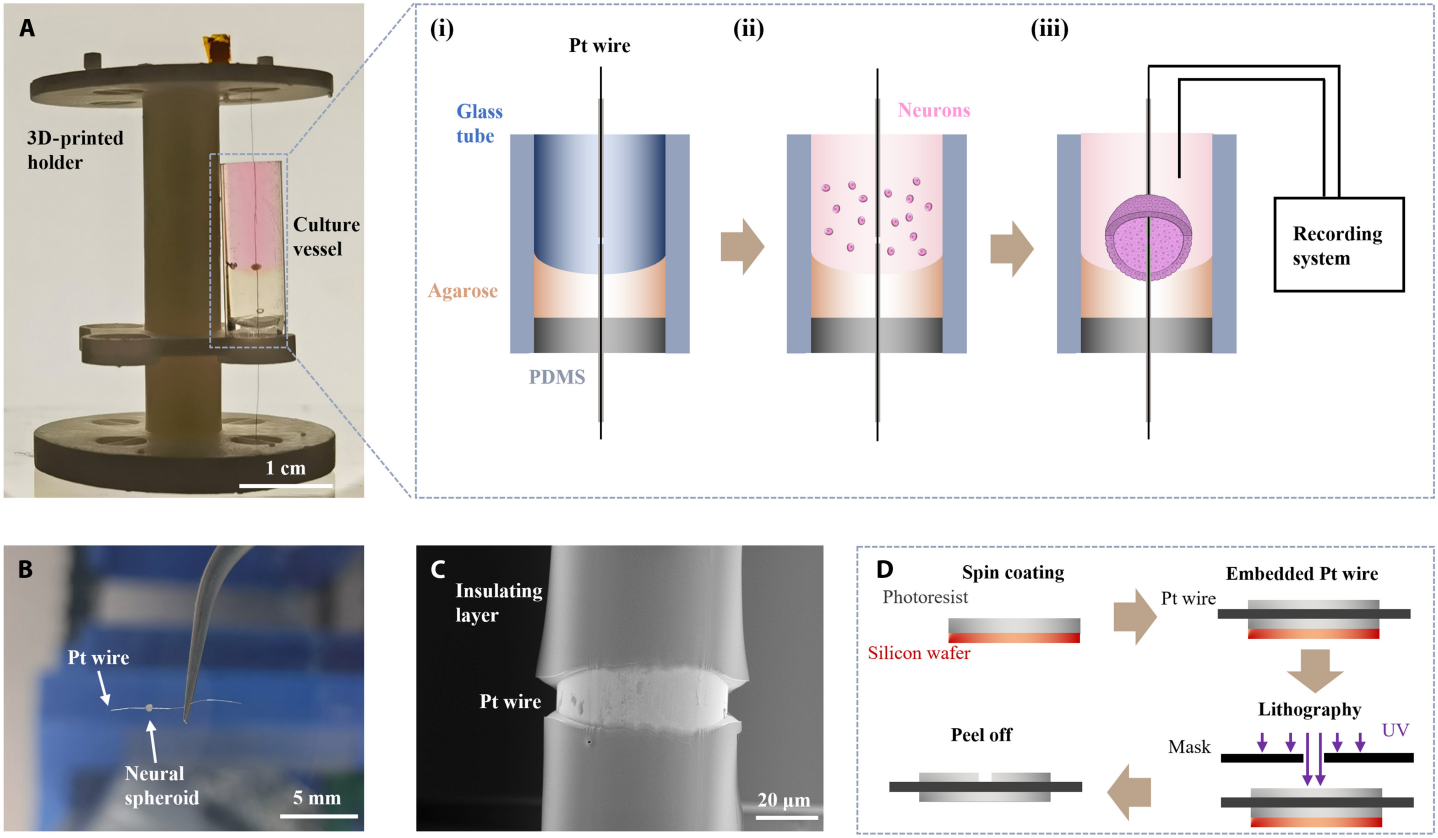
The wire-embedded device for forming lollipop-shaped neural spheroids. (A) Image of the 3D culture device, consisting of a 3D-printed holder and a single culture vessel. (i) Detailed view of the culture vessel structure. (ii) Neurons introduced into the culture vessel along with the medium. (iii) Formation of a neural spheroid with an inserted Pt wire, enabling the recording of interior signals of a neural spheroid through a recording system noninvasively. (B) Photograph of a neural spheroid cultured around a Pt wire. (C) SEM image of the inserted Pt wire, which was insulated by photoresist except for a 20-μm gap exposed for signal recording, to minimize noise interference. (D) Schematic illustrating the fabrication process of the insulated Pt wire.

The customized culture device comprising a 3D-printed holder, glass tubes, and Pt wires was introduced to generate neural spheroids with Pt wire inserted (Fig. 2A). The holder was designed to support up to 4 culture vessels, each critical for neural spheroid formation [Fig. 2A(i)]. Engineering drawings of the holder were shown in Fig. S1. To prevent medium leakage, a small piece of polydimethylsiloxane (PDMS) was strategically positioned at the base of each vessel. Agarose, offering a surface with low adhesive properties, served as the substrate for neural spheroid fabrication. A conductive wire was inserted straight through the center of the vessel to enable neural signal recording. Detailed fabrication process of the culture vessel was depicted in Fig. S2. To determine the optimal material for insertion, we rigorously evaluated the performance of Au, Pt, and tungsten (W) wires. Notably, W wire exhibited excessive rigidity, rendering it challenging to securely anchor within our device. Conversely, Au wire was discovered to be overly pliable and susceptible to deformation and was unable to maintain a straight alignment at the center of the device, while Pt wire, which offers an ideal equilibrium between rigidity and flexibility, perfectly aligned with our device’s requirements. To prevent wire loosening, excess thread was wound around the upper cylinder, ensuring a secure connection with the neural spheroid.

Neurons were subsequently introduced into the culture vessel [Fig. 2A(ii)], and within approximately 3 d, the low-adherence agarose promoted the formation of neural spheroids [Fig. 2A(iii)]. Following a 2-week maturation period, the neurons began producing signals. The neural spheroid was connected to a neural signal recording system by immersing the ground electrode in the medium, allowing for real-time interior recording. The Pt wire, securely attached to the neural spheroid, allows for precise signal capture. Upon maturity, the Pt wire could be cleanly severed at its base, allowing for the neural spheroid to be gently removed from the vessel (Fig. 2B). However, exposing the neural spheroids to air posed a risk of neuronal damage and reduced cell survival. Additionally, surface tension could cause displacement between the neural spheroid and the electrode filament when lifting the spheroid out of the aqueous environment. To enhance the effectiveness of neural spheroid transplantation, we discovered that submerging the entire vessel in a culture dish and carefully extracting the neural spheroid yielded superior outcomes.

In our device, 3 distinct materials come into contact with neurons: Pt, photoresist, and agarose. To ascertain their compatibility for neuronal culture, we individually coated Pt, photoresist, and agarose onto separate sections of a culture dish and subsequently cultivated neurons on these surfaces. The results of this experimentation are presented in Fig. S3, offering valuable insights into the interactions between these materials and neuronal cells. Specifically, Fig. S3A and B illustrates healthy neuronal growth on both the coated and uncoated sides, clearly demonstrating the minimal toxicity of Pt and photoresist toward NCs. Notably, as the cells are unable to adhere to the agarose coating, they migrate toward the uncoated side and continue to thrive (Fig. S3C). This observation not only validates the functionality of agarose in our setup but also confirms its low toxicity in this context.

To improve interior signal recording from the neural spheroid and minimize noise interference, we employed standard lithography techniques to coat the Pt wire with an insulating layer, meticulously leaving a 20-μm gap exposed for signal recording (Fig. 2C). The process involved spin coating a layer of photoresist on a silicon wafer, attaching the Pt wire to the wafer, and then applying another layer of photoresist on the wire. Lithography was used to expose a small gap in the Pt wire, which was then developed using a developer solution. The insulated Pt wire was subsequently assembled into the device, enabling signal recording within the neural spheroid (Fig. 2D).

**Fig. 3.**
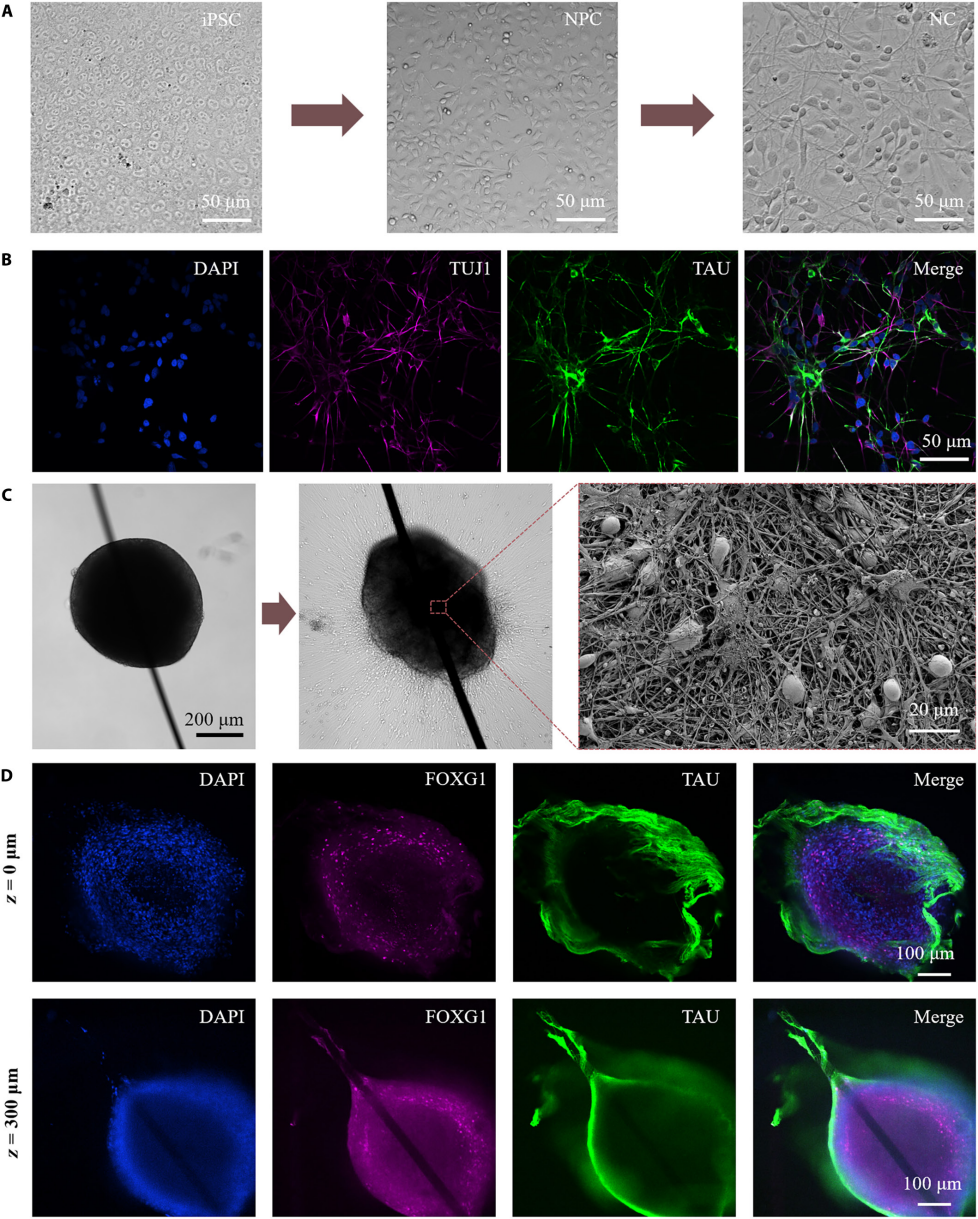
Immunofluorescence-based characterization of NCs and spheroids. (A) Schematic of the differentiation process from iPSCs to NPCs and subsequently to NCs. Each differentiation stage spans approximately 3 weeks. (B) Immunofluorescence staining of neurons. Visualizing the morphology of neurite outgrowth, with markers for TUJ1 in purple, TAU in green, and DAPI in blue, confirmed the maturation of neurons. (C) Neural spheroid grew on the petri dishes, and hundreds of axons spread around it. The surface of neural spheroid was observed by SEM (right). (D) Immunofluorescence staining of neurons in neural spheroids on planes of different heights, where *z* = 0 μm is the bottom of spheroid and *z* = 300 μm is the middle of the spheroid that can observe Pt wire clearly. Visualizing the morphology of neurite outgrowth with markers for DAPI in blue, FOXG1 in purple, and TAU in green, confirming the structure of neural spheroid.

Integration and coordination of neural circuits, which mimicked different cortical regions in humans, were investigated by using hiPSC (Fig. 3A). The differentiation process involved transforming hiPSCs into NPCs and subsequently into mature neurons. We confirmed the neuron maturation by staining with TUJI and TAU as neuron-specific cytoskeletal protein markers, and DAPI as a standard cell nucleolus marker, to visualize the cell nuclei for staining (Fig. 3B).

### Validation of neural spheroids

The biological structure of neural spheroids was accessed by transplanting the spheres into petri dishes for routine cultivation. After a 2-week period, the neural spheroid exhibited robust growth, characterized by the extension of numerous axons surrounding it, providing structural support (Fig. 3C). To gain further insights into its composition, we immobilized the spheroid using a fixative and stained it with TAU and FOXG1 (forkhead box G1, 1:1,000, Cell Signaling Technology, USA), which served as specific neuro-markers. Subsequently, the stained spheroid was mounted onto a confocal microscope slide for detailed examination under a confocal microscope (Fig. 3D). Near the bottom of the spheroid, green-stained floating axons were observed, indicating the healthy maturation of nerve cells within the spheroid. In the central region of the neural spheroid, the Pt wire was clearly discernible, passing seamlessly through the nerve sphere and integrating well with it. Remarkably, at the end of the neural spheroid, the axon wound around the Pt wire twice, establishing a stable connection and further enhancing the stability of neural signal acquisition.

Simultaneously, we aimed to validate the structural integrity of the neural spheroid through additional characterization. The spheroid was fixed, dehydrated, and coated before its surface was examined using SEM (Fig. 3C). The intricate and seemingly boundless nature of the surface axons showcased the complexity of the 3D cellular structure, thereby emphasizing the neural spheroid’s potential as a valuable model for neuron research.

### Investigation of the neural spheroid diameter

**Fig. 4.**
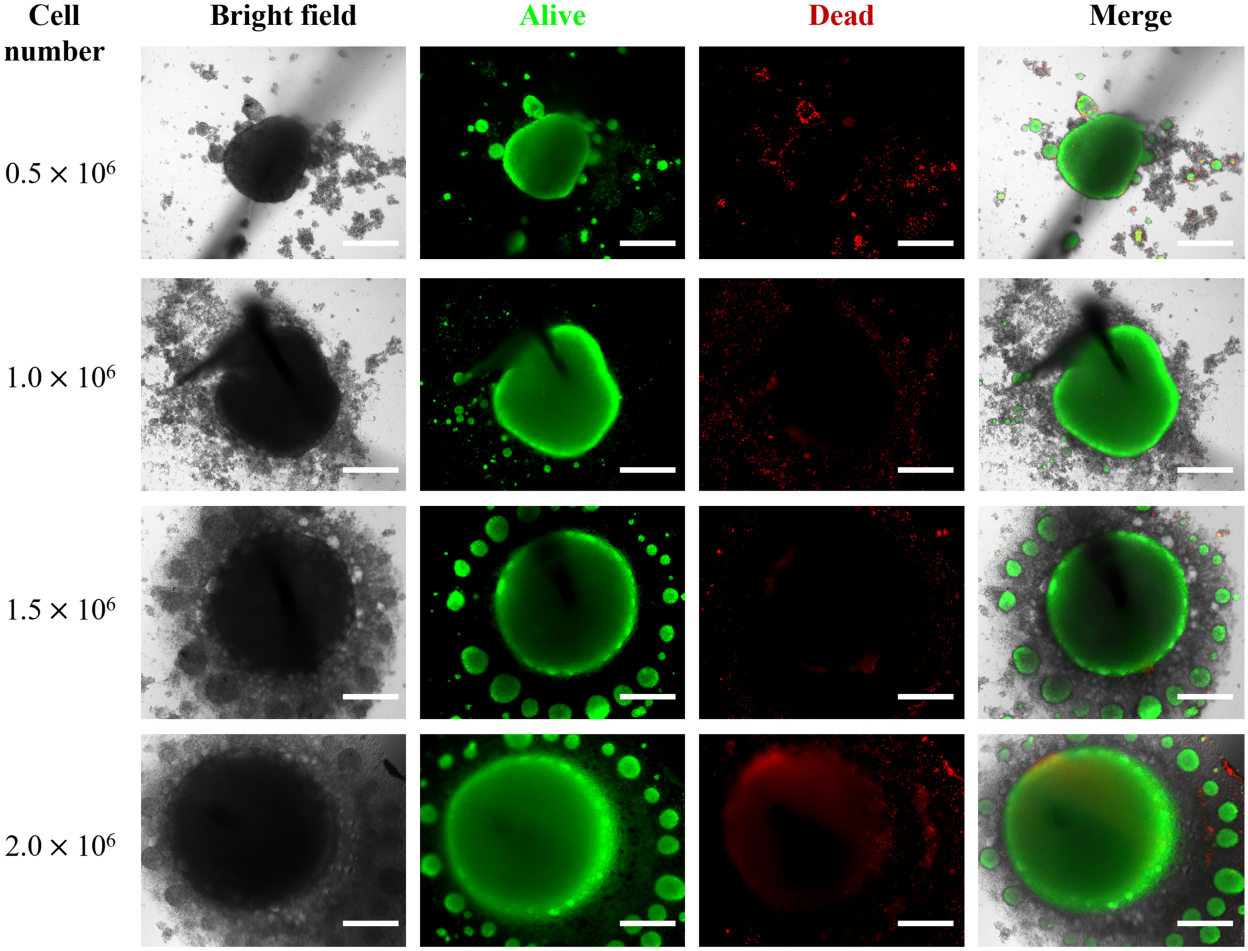
Neural spheroids at different diameters with viability tests (scale bar, 200 μm). Calcein-AM/PI staining kit was used to characterize cell viability, where live cells showed green color and dead cells showed red color. When cell number is lower than 1 million, the diameter of the neural spheroids increases with the cell increase; when cell number is larger than 1 million, the diameter of the spheroid was limited at 0.5 mm and the extra cells formed small aggregates surrounding the main spheroid.

Correlation between cell density and spheroid diameter was studied by embedding varying quantities of neurons (0.5, 1.0, 1.5, and 2.0 million) within the culture vessel, as illustrated in Fig. 4. Initially, we noted a proportional increase in the diameter of the neural spheroids with an increasing number of cells. However, upon reaching a cell count exceeding 1 million, we observed a plateau in the diameter growth, accompanied by the formation of small aggregates surrounding the spheroids with extra cells. Consequently, our findings indicated that the maximum achievable diameter of the neural spheroids within our culture device is approximately 0.5 mm, with the optimal cell number being 1 million. To assess the viability of the neural spheroids, we employed a commercial calcein-AM/PI staining kit, which allows for the simultaneous observation of both living and dead cells in the same cell culture dish under a fluorescence microscope. Calcein-AM can pass through the cell membrane and remove the AM group through the esterase action in living cells, and the resulting calcein emits strong green fluorescence. Therefore, the green fluorescence can be detected in living cells under fluorescence microscopy. PI (propyl iodide) can enter the dead cell through the damaged cell membrane and embed the DNA double helix to emit red fluorescence, so the dead cell will be detected with red fluorescence. The results indicated that the majority of neurons within the spheroids exhibited viability, thereby confirming the feasibility of our device for culturing neural spheroids with inserted Pt wire.

### Recording signals from neural spheroids

**Fig. 5.**
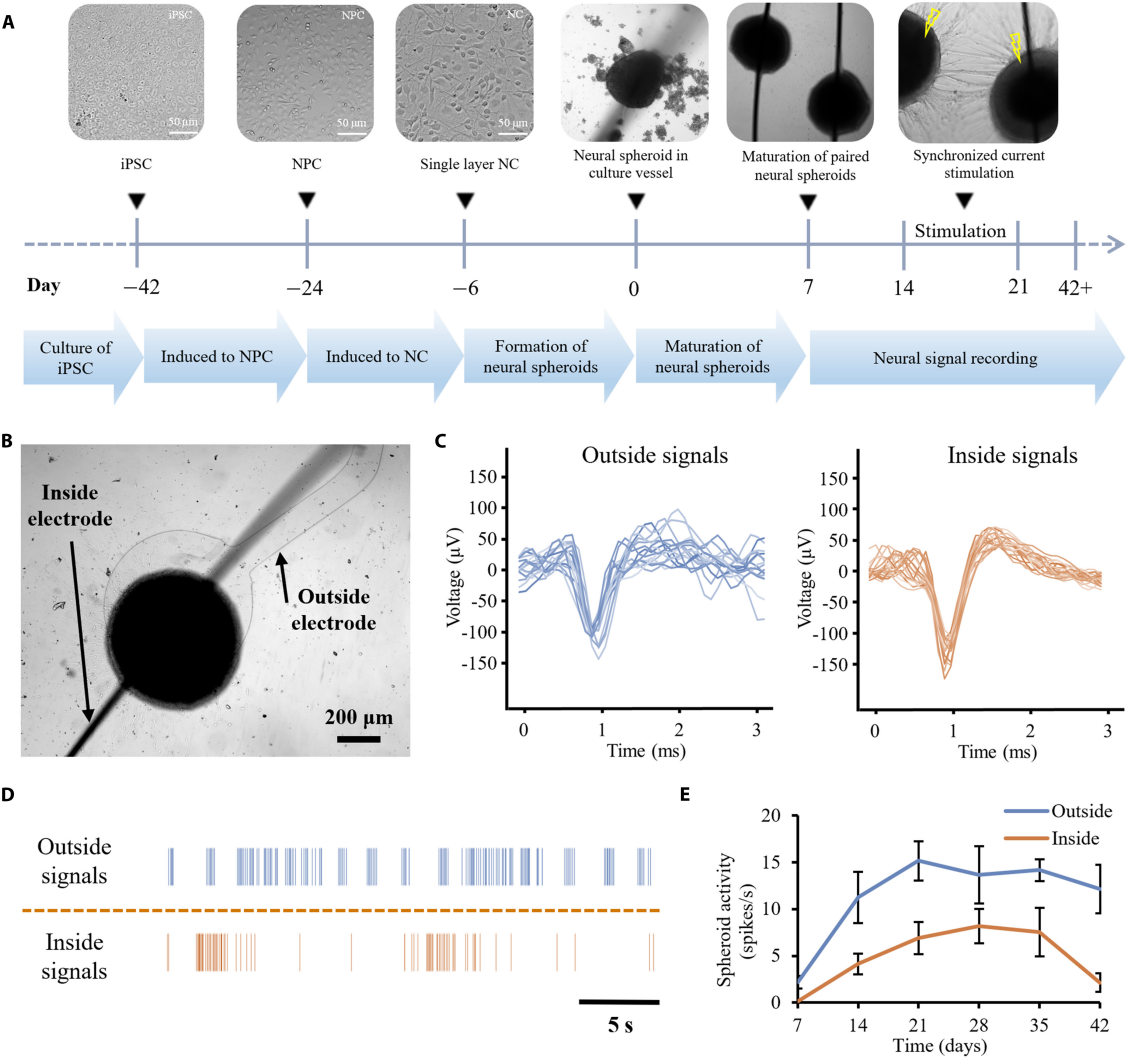
Signal recordings from the interior and exterior of the neural spheroid. (A) Timeline of neuron spheroid culture and neural signal recording operation. (B) Lollipop-shaped neural spheroid cultured on a 2D electrode over a period of 7 d. (C) Typical overlaid spike waveform showing spontaneous activity from both the interior and exterior of the spheroid. (D) Typical raster plot depicting spontaneous firing from the interior and exterior of the spheroid. (E) Spike rates recorded from the interior and exterior of the neural spheroid across different days.

The timeline of neurons and neural spheroid culture was shown in Fig. 5A. To capture neural signals within the neural spheroids, we connected the Pt wire to a data acquisition system while positioning ground electrodes in the culture medium adjacent to the spheroids. For recording external signals, neural spheroids were embedded on a glass plate with a transparent electrode (Fig. 5B). The electrode design incorporated a central exposed circle with a diameter of 20 μm, allowing for direct contact with neurons, while the surrounding area was insulated by photoresist to minimize noise interference. Fragments of the original neural signals from both the interior and exterior regions of the neural spheroid on day 21 are depicted in Fig. 5C. The results revealed that the spikes detected from the exterior cells exhibited greater noise levels, possibly due to environmental interference. Moreover, the average amplitude of signals recorded from exterior cells was slightly lower than those recorded from the interior cells, indicating higher signal quality within the spheroid.

Spontaneous activity behavior serves as a crucial indicator of neuron maturity and health [30]. To delve deeper into the spontaneous activity of neural spheroids, a raster plot of spikes over a half-minute interval on day 21 was generated (Fig. 5D). The results demonstrated that the exterior signals exhibited higher activity and frequency compared to the interior signals, which showed reduced activity and longer dormant periods. To quantify these observations, we calculated the spike rates from both interior and exterior of the neural spheroid over 5-min interval on a weekly basis, as depicted in Fig. 5E. Throughout the entire growth process, the spike rates within the spheroid were obviously much lower than those recorded outside. This discrepancy could be attributed to nutrient diffusion limitations within the neural spheroid, potentially causing neurons to have restricted access to nutrients, which in turn results in less frequent neuronal stimulation.

### Investigation of the interconnection between neural spheroids

**Fig. 6.**
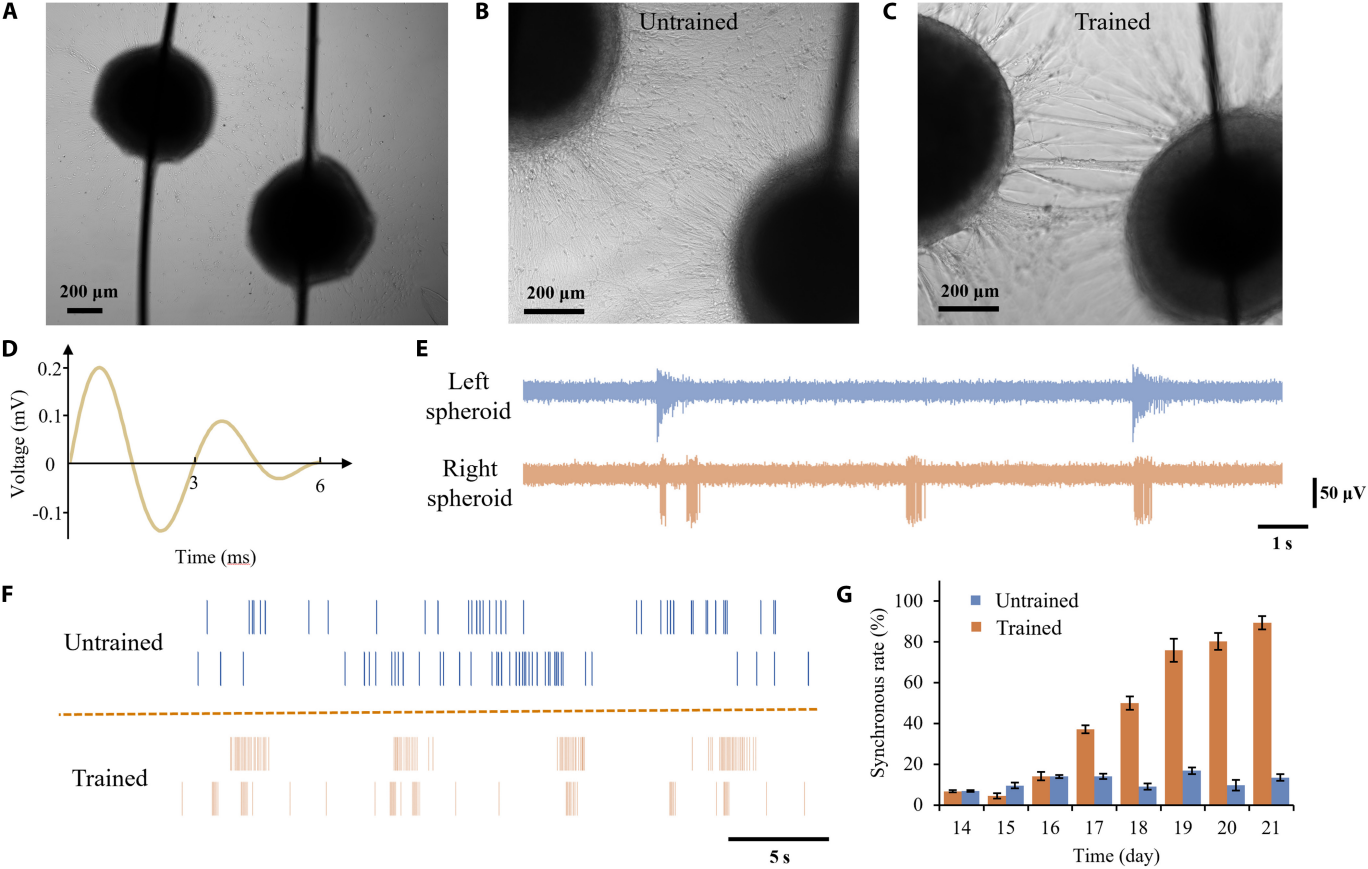
Interconnection and synchronization between neural spheroids. (A) Two lollipop-shaped neural spheroids positioned in close proximity, each extending axons toward the other. (B) Axon connection between 2 neural spheroids without training, showing disorganized and sparse axonal. (C) Axon connection between 2 neural spheroids with training, demonstrating focused and robust neural tract formation. (D) Stimulation protocol: A fading sinusoidal signal with an amplitude of 0.2 mV and a period of 3 s applied to the neural spheroids. (E) Original neural signals recorded from the 2 trained neural spheroids, illustrating the quality of interior recordings. (F) Raster plot comparing spontaneous firing patterns of the neural spheroids with and without training, revealing synchronized activity in the trained group. (G) Synchronous firing rates of the neural spheroids measured on day 14 (start of the training) and subsequent days, showing enhanced synchronization with training.

Interspheroids connection is an excellent model to study the neural connection across different cortical regions, which was achieved by placing 2 spheroids in close proximity to each other on a culture dish, as shown in Fig. 6A. Over time, the spheroids exhibited axonal growth, firmly anchoring themselves and establishing connections with one another. As depicted in Fig. 6B, the axons gradually became denser and formed tighter connections between the spheroids. To further enhance the interconnection between them, we conducted an additional set of experiments involving synchronized current training. According to the results in Fig. 5E, neural signals originating from the exterior of the spheroids commenced on day 7, whereas interior signals initiated on day 14 and attained stability by day 21. This pattern suggests that neurons undergo a gradual maturation process within the first 21 d to establish neural networks. Consequently, we selected the period from day 14, when interior neurons began generating signals, to day 21, when their signals became stable, to enhance the connections between neural spheroids. The spheroids received synchronized currents twice per minute for 10 min daily. After 1 week of training, a remarkable consolidation of interconnections between the spheroids was observed, resulting in the formation of a robust neural tract, as illustrated in Fig. 6C.

A fading sinusoidal signal was generated to imitate real neural signals and stimulate the neural spheroids, as shown in Fig. 6D. To assess the neural spheroids’ voltage tolerance, we employed sinusoidal signals of varying amplitudes for daily 10-min stimulation sessions. The impact of these stimulations on the neural spheroids is illustrated in Fig. S4, revealing a critical threshold. Specifically, amplitudes exceeding 20 mV resulted in irreversible neuronal damage, while those above 2 mV posed a risk of potential neuronal harm, thereby compromising the longevity of the spheroids. Conversely, amplitudes below 2 mV were deemed relatively safe, exhibiting negligible effects on the spheroids. To ensure the safety of our neural spheroids and achieve a more realistic simulation of neural signals, we opted for a voltage amplitude of 0.2 mV, administered in 3-s intervals. This amplitude closely resembles the spikes naturally detected within the neural spheroids, thereby optimizing the experimental conditions for both safety and physiological relevance.

We connected the trained neural spheroids to the recording system, and the original neural signals displayed in Fig. 6E revealed synchronized bursts of activity from the 2 neural spheroids in the trained group. Additionally, the raster plot of neural signals (Fig. 6F) demonstrated synchronized activity between the spheroids. To quantify the level of synchronization between the 2 neural spheroids, we calculated the synchronous rate with and without training from day 14 to day 21, as depicted in Fig. 6G. The results indicated a rapid increase in the synchronous rate after 3 d of synchronized current stimulation. These findings highlight the efficacy of synchronized current stimulation in enhancing interconnections between neural spheroids and promoting synchronization. This provides valuable insights for training neural networks in vitro.

The application of synchronized current stimulation altered the properties of neural spheroid connections, thereby enhancing our neural tract model. First, there is a notable difference in spontaneous activity; trained neural spheroids exhibit more structured and synchronized spontaneous activity due to the strengthened interconnections and synchronization achieved through this stimulation. Second, the morphological development of axons within these trained spheroids demonstrates a significant enhancement of interconnections, resulting in robust neural tracts that facilitate more effective neuronal communication. The axons between untrained neural spheroids are attached to the surface of the petri dish. Lastly, neural spheroids subjected to training display an improved capacity to respond to external stimuli, exhibiting synchronized bursts of activity in response to electrical signals that mimic real neural activity. These findings offer valuable insights into the potential of in vitro neural network training and present promising implications for future research in neural spheroid and brain organoid science.

## Discussion

In this study, a novel wire-inserted cell culture device for noninvasive recording signals from inside NCs of spheroids was proposed, enabling the systematic investigation of their structural and functional properties. While our culturing device for neural spheroids offers numerous advantages and insights, it is important to acknowledge its limitations. First, the maximum achievable diameter of the neural spheroids within the culture device is approximately 0.5 mm. This size restriction may limit the complexity and functionality of the neural networks that can be studied [31]. Scaling up the culture system to accommodate larger spheroids or multiple interconnected spheroids may pose technical challenges. Second, the culture device provides a controlled and simplified environment for the neural spheroids. While this allows for precise manipulation and observation, it may not fully capture the complexity and dynamic nature of in vivo neural networks [32,33]. Factors such as immune responses, blood supply, and interactions with neighboring tissues are absent in this simplified in vitro system. At last, the number of recording electrodes was limited due to the scale of the Pt wire. It would be better to replace Pt wire by some integrated needle-shaped complementary metal oxide semiconductor (CMOS) electrodes to have spatial resolution inside the neural spheroids [34]. Understanding and addressing these limitations will be crucial for further advancements in the field of neural spheroid research and the development of more sophisticated and physiologically relevant in vitro models.

**Fig. 7.**
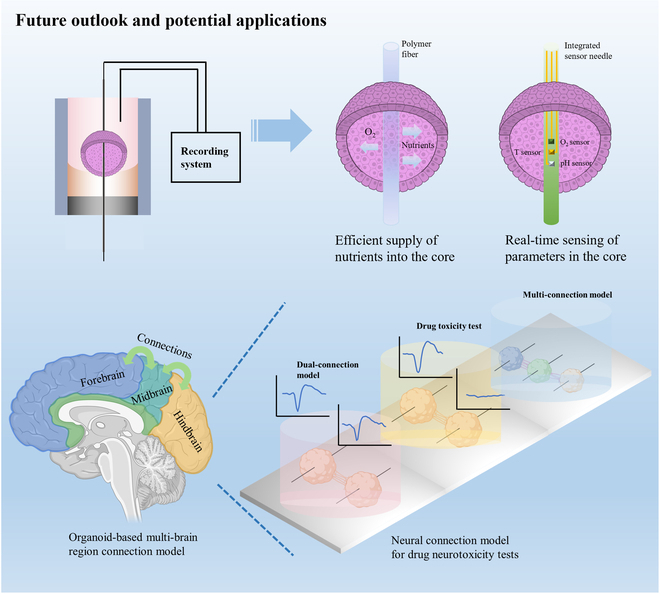
Potential applications of the wire-embedded 3D neural spheroid culture device. First, replacing Pt wire with a porous polymer fiber could enable oxygen and nutrients to permeate the neural spheroids or brain organoids, potentially alleviating central hypoxia. Alternatively, using functional needles instead of Pt wires could allow for the integration of multi-needle sensors to monitor the interior conditions such as O_2_, pH, and temperature simultaneously in real time. Second, the wire-embedded 3D culture system can model connections between different brain regions using organoids and support drug toxicity tests of potential neurological therapies.

Based on the findings and limitations of our study, there are several promising directions for further advancing this work. First, exploring various materials as center wires could offer different functionalities. For instance, substituting Pt wire with porous polymer fiber could enhance the permeation of oxygen and nutrients into the neural spheroids, effectively addressing the issue of central hypoxia. Various aspects of neural spheroid culture and connection possess the potential for further improvement. Theoretically, integrating polymer fiber within neural spheroids could bolster the supply of oxygen and nutrients, thus stimulating more vigorous neural activity. Additionally, these improved conditions may also foster better synchronization between neural spheroids. Alternatively, replacing the Pt wire by a needle, integrated with sensors, would enable real-time monitoring of the conditions inside the neural spheroids. Another potential avenue is to culture brain organoids, which closely resemble the structure of the human brain, in the developed device [35,36]. In our device, there exist notable differences between culture neural spheroids and brain organoids, primarily in the initial embedding process, where stem cells are utilized instead of NCs. Subsequently, different culture medium was added according to the organoid culture protocol to differentiate these stem cell spheroids into forebrain, midbrain, or hindbrain organoids. This would enable the recording of interior signals within the organoids, mimicking the complex interactions and supportive functions observed in vivo [25]. By culturing different brain organoids together, it would be possible to study the connections between different brain regions, as illustrated in Fig. 7. Three types of brain organoids were placed in close proximity to facilitate robust neural tract connections, mimicking the interconnections between various human brain regions. This approach will enhance our comprehension of network behavior and communication between brain regions. Additionally, integrating advanced stimulation techniques such as optogenetics or electrical stimulation protocols that replicate specific spatiotemporal patterns observed in vivo could provide precise and targeted manipulation of neural activity within the spheroids [37,38]. Furthermore, utilizing the developed culture device and neural spheroid/organoid models for studying neurological disorders and drug screening holds great promise. Epilepsy serves as an example, characterized by abnormal neuronal firing in the brain, ultimately causing brief electrical disturbances that may lead to acute confusion or convulsion. Depending on the affected neuronal parts, this firing can be localized or widespread. By chemically inducing the formation of an epilepsy brain organoid and connecting it with a normal brain organoid, we can observe the transmission of the disease from one organoid to another. Furthermore, we can test the efficacy of drugs in the treatment of epilepsy or mitigating the transmission of epilepsy. This approach could provide valuable insights into the mechanisms underlying these disorders and facilitate the discovery of novel therapeutic approaches. By pursuing these future directions, we can advance the field of neural spheroid and brain organoid research, deepen our understanding of neural network dynamics, and potentially uncover innovative therapies for neurological disorders.

## Conclusion

We have presented a novel wire-inserted cell culture device that substantially elevates the capacity to noninvasively record signals from within neural spheroids. This approach not only permits a systematic examination of structural and functional properties of neural spheroids but also exhibits numerous advantages over existing methods. Our device fosters the successful cultivation and integration of axons within the spheroids, establishing robust interconnections between paired neural spheroids. This represents a pivotal advancement in modeling intricate neural networks and elucidating their dynamics.

A key advantage lies in the superior signal quality obtained from interior recordings compared to outside recordings. This is particularly valuable for studying neural activity deep within the spheroids, where conventional methods often struggle to achieve reliable recordings due to potential minor injuries. Furthermore, the synchronized current stimulation we employed further enhanced interconnection and synchronization between spheroids, demonstrating the potential of our device to manipulate and modulate neural networks. An additional notable advantage is the capability to precisely manipulate the spheroids using the inserted wire. This allows us to position spheroids onto MEA chips for comparative analysis of neural signals within and outside the spheroids. Such comparative analysis is invaluable for unraveling the influence of the spheroid microenvironment on neural activity and connectivity. Understanding interspheroidal connections is crucial for comprehending information transfer and synchronization mechanisms in neural networks, which are fundamental to both healthy and diseased states. Moreover, the versatility of our device permits extensive customization and expansion. For instance, replacing the Pt wire with polymer fibers could potentially supply nutrients into the center of neural spheroids, significantly enhancing their survival rates. This underscores the capacity to tailor the device to address specific research needs and optimize neural spheroid cultures.

In summary, our novel wire-inserted cell culture device presents several advantages over existing methods, facilitating superior signal quality from interior recordings and precise manipulation of neural spheroids. Its potential applications in neurobiology and neural network studies, along with its versatility for customization and scalability, position it as a promising and versatile tool for advancing the understanding of neural network dynamics and uncovering novel therapeutic approaches for neurological disorders.

## Data Availability

The data that support the findings of this study are available from the corresponding author upon reasonable request.
