# Supplementary material for: Platinum Wire-Embedded Culturing Device for Interior Signal Recording from Lollipop-Shaped Neural Spheroids
Source: Cyborg Bionic Syst. 2025 Mar 5;6:0220. doi: 10.34133/cbsystems.0220 (PMC11880574; doi:10.34133/cbsystems.0220)
Supplement: Supplementary 1 — Figs. S1 to S4 [file cbsystems.0220.f1.pdf]

Supplementary Materials for

Platinum Wire-embedded Culturing Device for Interior Signals Recording from Lollipop-shaped Neural Spheroids

Hongyong Zhang[1], Nan Huang[2], Sumin Bian[1,*], and Mohamad Sawan[1,*]

[1]CenBRAIN Neurotech, School of Engineering, Westlake University, Hangzhou, Zhejiang 310030, China
[2]School of Life Science, Westlake University, Hangzhou, Zhejiang 310030, China
**Corresponding authors: biansumin@westlake.edu.cn; sawan@westlake.edu.cn*

Supplemental information includes four figures.

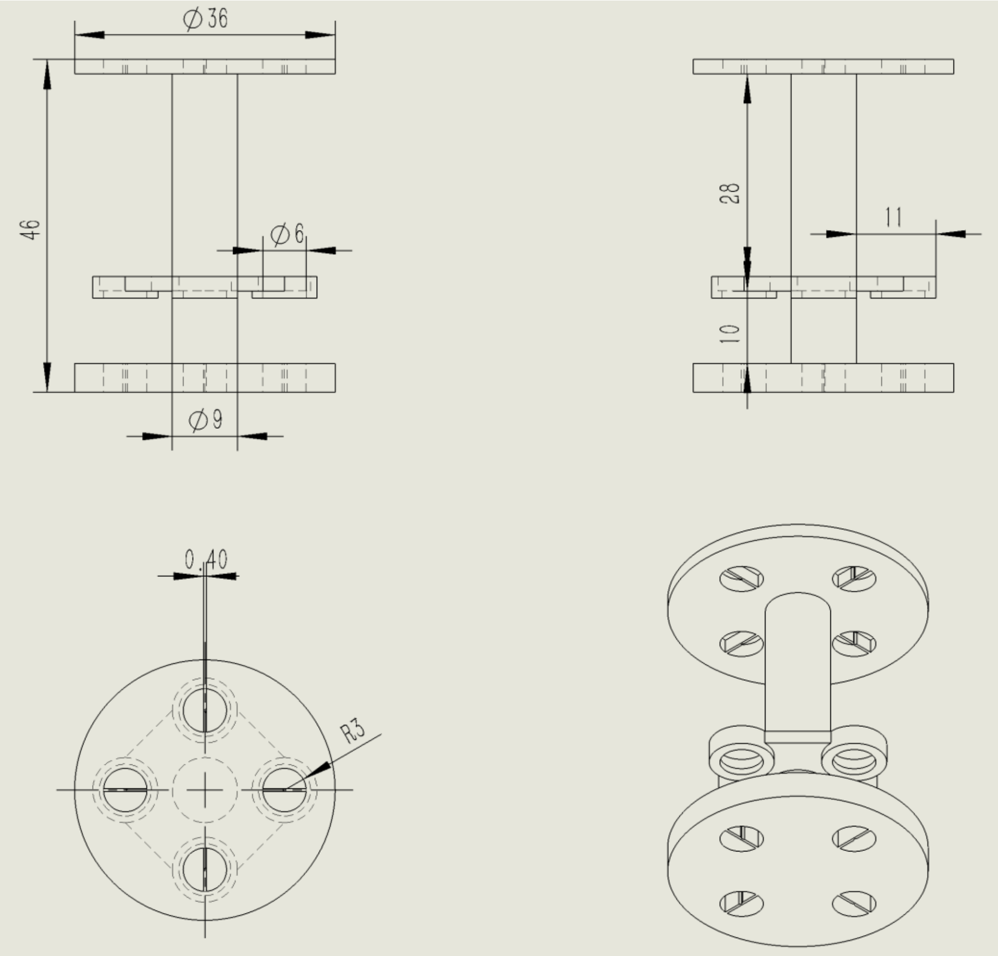

Fig. S1. Engineering drawings of the 3D printed holder.

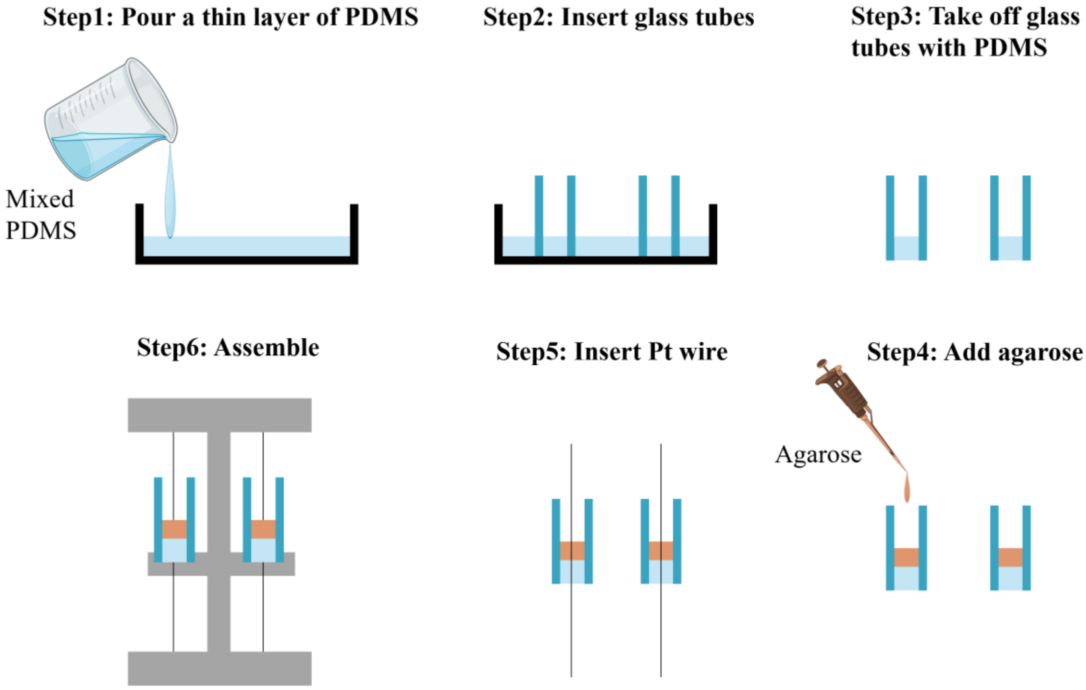

Fig. S2. Fabrication process of the culture vessel units.

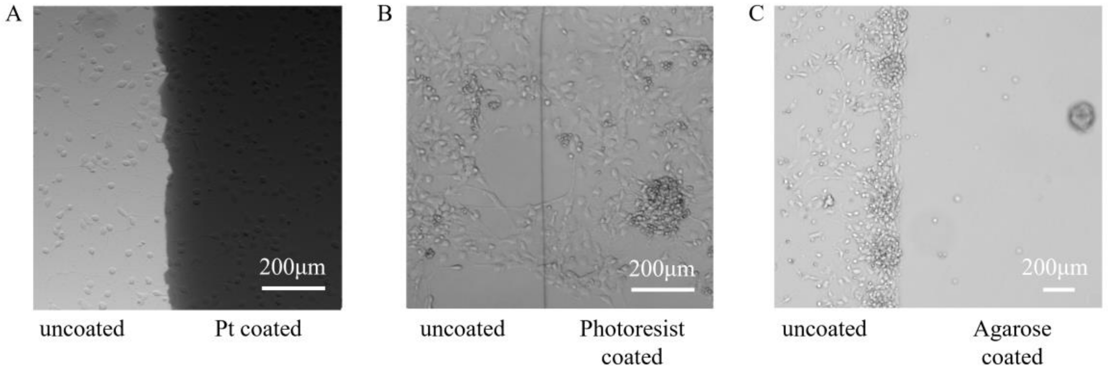

Fig. S3. Neural toxicity tests of the materials in our device: (A) Neurons cultured on dish with half side coated by Pt; (B) Neurons cultured on dish with half side coated by photoresist; (C) Neurons cultured on dish with half side coated by agarose. Neurons migrate towards the uncoated side and continue to thrive, thereby validating not only the functionality but also the low toxicity of agarose.

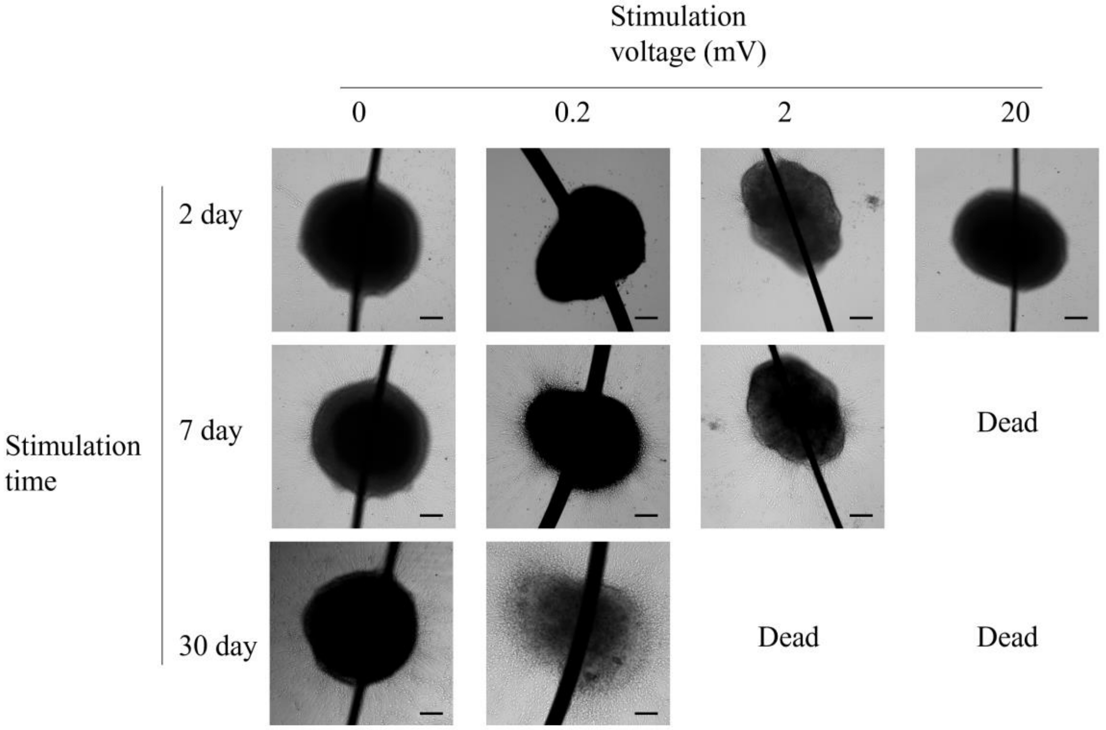

Fig. S4. The state of neural spheroids under various stimulation voltages, observed across distinct time intervals, undergoes a dynamic growth progression. Specifically, amplitudes exceeding 20 mV resulted in irreversible neuronal damage, while those above 2 mV posed a risk of potential neuronal harm, thereby compromising the longevity of the spheroids. Conversely, amplitudes below 2 mV were deemed relatively safe, exhibiting negligible effects on the spheroids (Scale bar = 100μm).
